# Determining the origin of different variants associated with familial mediterranean fever by machine-learning

**DOI:** 10.1038/s41598-022-19538-1

**Published:** 2022-09-08

**Authors:** Orit Adato, Ronen Brenner, Avi Levy, Yael Shinar, Asaf Shemer, Shalem Dvir, Ilan Ben-Zvi, Avi Livneh, Ron Unger, Shaye Kivity

**Affiliations:** 1 The Mina and Everard Goodman Faculty of Life Sciences, Bar-Ilan University, Ramat-Gan, Israel; 2 Institute of Oncology, Wolfson Medical Center, Holon, Israel; 3 Sackler Medical School, Tel Aviv University, Tel-Aviv, Israel; 4 Israel Heller Institute of Medical Research, Sheba Medical Center, Tel Hashomer, Israel; 5 Department of Medicine B, Assuta Ashdod Medical Center, Ashdod, Israel; 6 Department of Ophthalmology, Shamir Medical Center (Formerly Assaf-Harofeh), Tzrifin, Israel; 7 Department of Medicine F, Sheba Medical Center, Tel-Hashomer, Israel; 8 Rheumatology Unit, Meir Medical Center, Cfar-Saba, Israel; 9 Department of Ophthalmology, Shamir Medical Center, 70300 Be’er Ya’akov, Israel

**Keywords:** Genetics, Immunology

## Abstract

A growing number of familial Mediterranean fever (FMF) patients in Israel do not have a single country of origin for all four grandparents. We aimed to predict the Mediterranean fever gene (*MEFV*) variant most likely to be found for an individual FMF patient, by a machine learning approach. This study was conducted at the Sheba Medical Center, a referral center for FMF in Israel. All Jewish referrals included in this study carried an FMF associated variant in *MEFV* as shown by genetic testing performed between 2001 and 2017. We introduced the term ‘origin score’ to capture the dose and different combinations of the grandparents’ origin. A machine learning approach was used to analyze the data. In a total of 1781 referrals included in this study, the p.Met694Val variant was the most common, and the variants p.Glu148Gln and p.Val726Ala second and third most common, respectively. Of 26 countries of origin analyzed, those that increased the likelihood of a referral to carry specific variants were identified in North Africa for p.Met694Val, Europe for p.Val726Ala, and west Asia for p.Glu148Gln. Fourteen of the studied countries did not show a highly probable variant. Based on our results, it is possible to describe an association between modern day origins of the three most common *MEFV* variant types and a geographical region. A strong geographic association could arise from positive selection of a specific *MEFV* variant conferring resistance to endemic infectious agents.

## Introduction

Familial Mediterranean fever (FMF) is the most common syndrome in the group of hereditary auto-inflammatory diseases^1^. It is an autosomal recessive disease that mainly associates with variants in the *MEFV* gene, located on chromosome 16. *MEFV* encodes the pyrin protein, which is important for the inflammatory response to infectious agents^2^. More than 300 variants of the *MEFV* gene have been identified in Infevers https://fmf.igh.cnrs.fr/ISSAID/infevers/search.php?n=1 (Infevers: an online database for autoinflammatory mutations. Copyright. Available at https://infevers.umai-montpellier.fr/ Accessed at (02/2022)^3–6^). The five most common variants (p.Met694Val, p.Val726Ala, p.Met694Ile, p.Met680Ile and p.Glu148Gln) account for the vast majority of cases^7,8^. The prevalence of FMF is highest among ethnic inhabitants of the Mediterranean basin with a carrier rate of up to 1 in 4 in certain populations. In recent years, the disease has been reported in ethnically heterogeneous patients around the globe^9–13^. Israel is considered an endemic area for FMF^14,15^. Its current population has diverse origins in the Jewish diaspora including Europe, northern Africa, and Asia. There is a correlation between the p.Met694Val variant and Jewish Moroccan ethnicity as well as with a severe disease phenotype^16,17^. However, associations between other countries of origin and FMF variants have been only partially established^18^. Such knowledge is important to understand the epidemiology of FMF. Here we use a novel approach, based on a machine learning algorithm, to predict the mutation type carried by a patient based on the countries of origin of his/her parents or grandparents.

## Subjects and methods

### Setting and patient selection

This study was conducted at The Chaim Sheba Medical Center in Tel Hashomer, Israel, which is a referral center for genetic testing and evaluation of FMF patients. First, we collected data on all referrals to our center for genetic analysis by their primary physician following a clinical suspicion for FMF between 2001 and 2017. All referrals negative for variants in *MEFV* were excluded. Since mixed origins mainly characterize Jewish patients only Jewish referrals were included in our study group. Data regarding the gender and the specific variant of each referral was extracted from medical records. This research was approved by Sheba Medical Center institutional ethics committee. All methods were performed in accordance with the relevant guidelines and regulations.

### Genetic analysis of *MEFV*

For the genetic analysis, DNA was extracted from 100 µl of blood taken from the referral using a Puregene kit (Gentra Inc.) and was screened for five known variants in *MEFV*, LRG190t1:c.2080A > G p.(Met694Val), c.2177 T > C p.Val726Ala, c.422G > C, p.Glu148Gln, c.20420G > A or c.2040G > C p.Met680Ile, and c.2082G > A p.Met694Ile, using a commercial kit (Gamidagen) or polymerase chain reaction (PCR) amplification and restriction enzyme analysis^19^.

### Computational analysis

#### Origin score

In genetic studies, it is usually straightforward to investigate the association between country of origin and variants using mathematical tools such as Bayes rule. However, given the ancestral diversity of the Israeli Jewish population, the subjects referred to our center often do not have a single country of origin. It was therefore necessary to construct a model using machine learning in order to perform statistical analysis. We included in the analysis countries from which at least 15 referrals originated. Based on this threshold, the data used for the analysis included 26 countries (out of 48 reported to be countries of origin for parents or grandparents by patients in the cohort). First, data on referrals and countries of origin were tabulated in a matrix with a row representing a subject and a column representing a possible country of origin. We then calculated an “Origin Score” in the following way: In each cell we stored the fraction of the subject’s origin from each country. For example, if a referral has two grandparents from Algeria, one from Morocco, and one from Iraq, then the values of the corresponding cells will be 0.5, 0.25, and 0.25, respectively, and cells corresponding to all other countries will be assigned a value of 0. If information about the country of origin of one of the grandparents was missing, it was assumed that both grandparents from that side had the same country of origin. Subjects without information on at least one grandparent from each side were excluded from the analysis. Based on the method described above, we calculated for every country, the sum of origin scores of subjects with any level of ancestry from that country. The sum of origin scores per country is presented in Fig. S1.

#### Machine learning approach

The logic behind our novel machine learning based approach is that the level at which we are able to predict if a person has a specific variant based on his/her origin is an indication of the strength of the correlation between the origin and the variant. Clearly, the stronger the association between a given country of origin and a specific variant the more accurate is the prediction. For the machine learning approach, we used the logistic regression module "scikit-learn” in Python 2.7^20^. Logistic regression is a linear model used to measure the relationship between the categorical dependent variable and one or more independent variables by estimating probabilities describing the possible outcomes based on the logistic function. In our study, we used the countries of origin as the independent variables and attempted to predict the specific variant as a categorical dependent variable (i.e., if the person has or does not have the specific variant). The performance of the prediction was evaluated by the area under the curve (AUC) measure, which shows the deviation of the performance from a random prediction, which has an AUC value of 0.5 while a perfect prediction has a value of 1. We validated our model using tenfold cross validation (dividing the data randomly each time to 90% for training and 10% for testing) and by bootstrapping (where subsets were resampled with replacement 1000 times and patients that were not included in the sample were used as the test dataset). For each prediction, we averaged each vector coefficients and used the result to identify origins that are positively and negatively associated with certain variants. Since our analysis revealed that the p.Met694Ile and p.Met680Ile variants were very rare in our study sample, we excluded these variants from the subsequent analysis. Therefore, we included in the final analysis only the three most common variant types: p.Met694Val, p.Val726Ala and p.Glu148Gln. The data used for the prediction of the country of origin included only patients that carry a single type of mutation, either homozygous or heterozygous. Compound heterozygotes were not included since we did not have enough data for each compound heterozygous pair.

#### Selection of country groups

We combined the different countries of origin into groups that contained four countries each, covering four possible origins per patient. We formed a group from the four countries that were ranked highest in their association with each variant and another group with the four countries that were ranked as the least associated.

### Ethics committee

The study has been approved by the appropriate ethics committee. Informed consent was waived by the ethics institutional review board (IRB) – Sheba Medical Center (SMC-9763-12).

## Results

A total of 1842 referrals for *MEFV* genetic testing had at least one *MEFV* gene variant. After excluding 61 subjects with uncommon variants, we included 1781 subjects (52% females) in our analysis (Table 1). The number of subjects detected with each *MEFV* variant is presented in Fig. S2. The p.Met694Val variant was found in 72% of the referrals. Out of the 1286 referrals that carry p.Met694Val, 18% (240) had another variant; these referrals were compound heterozygous for either p.Glu148Gln, p.Val726Ala, p.Met680Ile, or p.Met694Ile. The prevalence of referrals with the p.Glu148Gln and p.Val726Ala variants were 23% (419 subjects) and 19% (342 subjects), respectively. Of the referrals that carried p.Glu148Gln, 35% (150) were compound heterozygous either with p.Met694Val, or p.Val726Ala. Of those with the p.Val726Ala variant, 42% (145) were compound heterozygous.

**Table 1:** Prevalence of common *MEFV* variants in Jewish FMF referrals to genetic testing between 2001 and 2017 in one referral center.

Variant, zygosity status	Patient number by gender		Total
	Female	Male	
p.Met694Val, (HTZ)	421	403	824
p.Glu148Gln, (HTZ)	142	119	261
p.Met694Val, (HMZ)	122	100	222
p.Val726Ala, (HTZ)	88	72	160
p.Met694Val and p.Glu148Gln, Compound heterozygous E148Q	58	63	121
p.Met694Val and p.Val726Ala, Compound heterozygous	49	67	116
p.Val726Ala, (HMZ)	16	21	37
p.Val726Ala and p.Glu148Gln, apparent compound heterozygous	15	14	29
p.Glu148Gln, (HMZ)	6	2	8
p.Met694Val and p.Met680Ile, Compound heterozygous	2		2
p.Met694Val and p.Met694Ile, Compound heterozygous	1		1
Total	920	861	1781

*HMZ* homozygote, *HTZ* heterozygote.

The results presented here are based on 10-Fold cross-validation (see Methods). Similar results were achieved using bootstrap resampling with replacement 1000 times (See supplementary Fig. S3).

**Figure 1:**
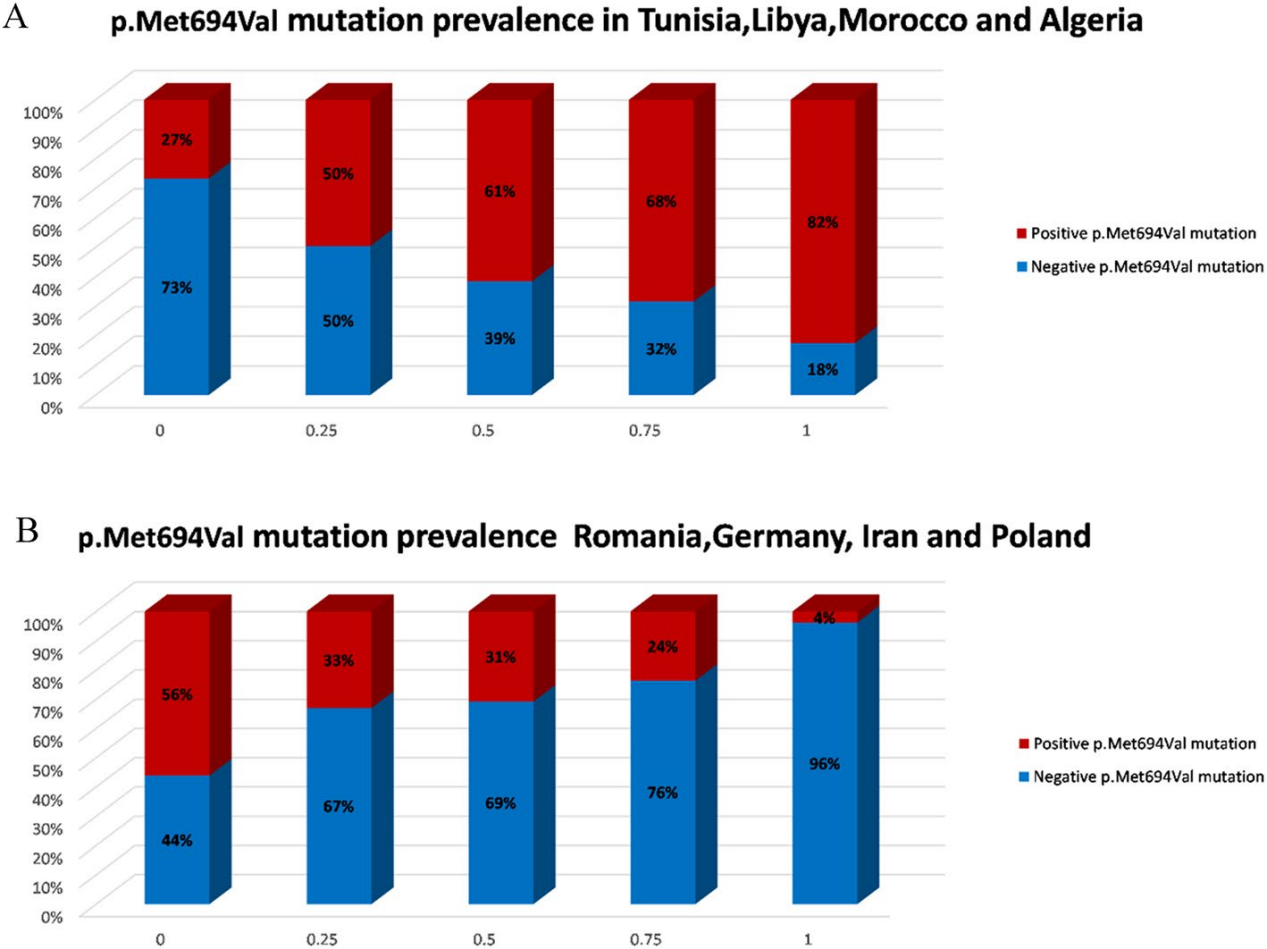
Prevalence of the countries of origin most indicative for the p.Met694Val variant: (**A**) Tunisia, Libya, Morocco, and Algeria are the countries of origin most positively associated with the p.Met694Val variant. (**B**) Romania, Germany, Iran and Poland are the countries of origin most negatively associated with the p.Met694Val variant. Red and blue bars indicate patients who carry and do not carry the p.Met694Val variant, respectively. The x-axis indicates the amount of grandparents from the four countries (0 being none, 1 being all four).

**Figure 2:**
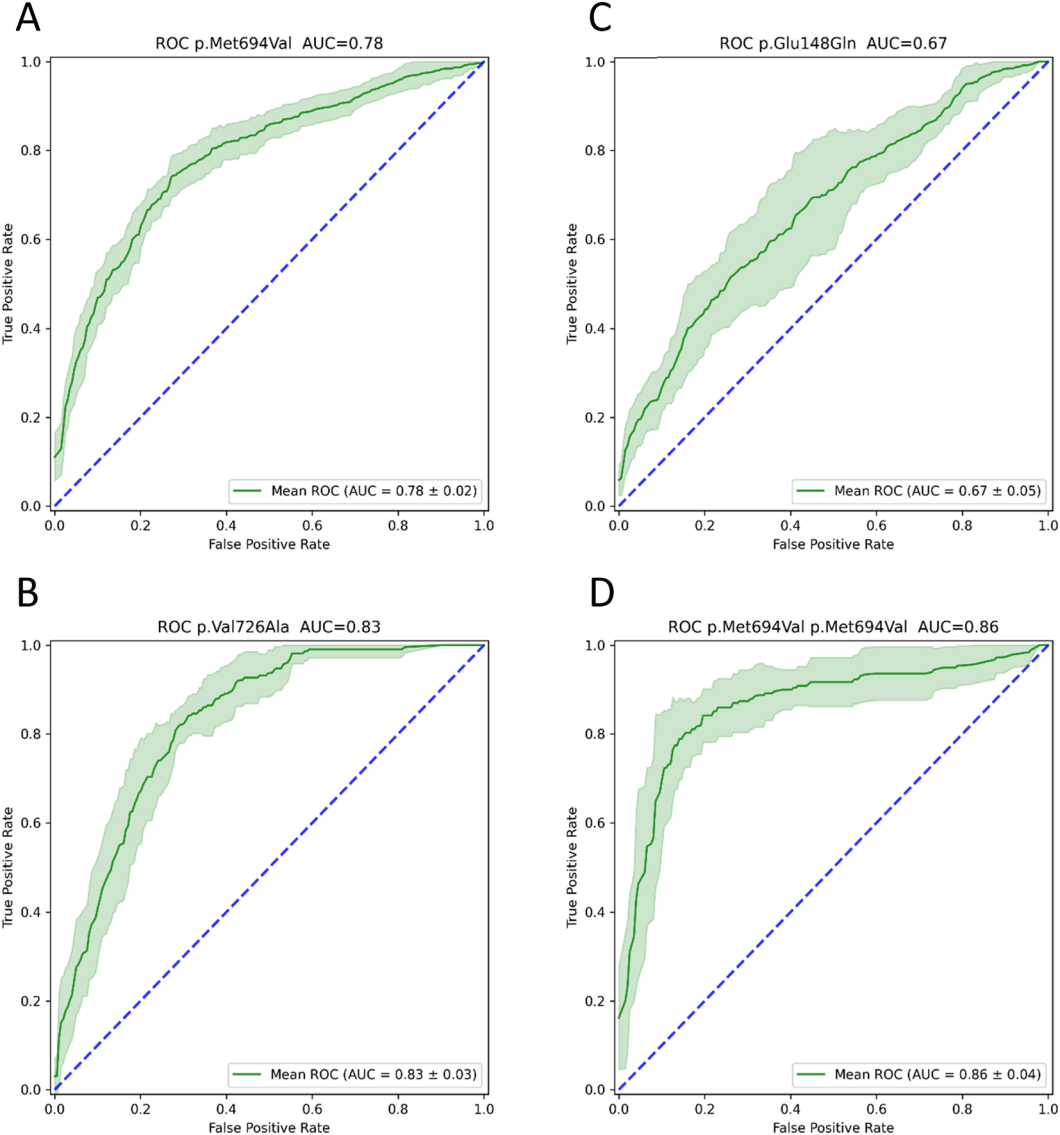
ROC of multivariate logistic regression variant prediction. The figure represents the performance of the multivariate logistic regression model that uses 27 features (26 countries and sex of the patient) to predict whether a patient: (**A**) carries the variant p.Met694Val, (**B**) carries the variant p.Val726Ala, (**C**) carries the variant p.Glu148Gln, and (**D**) is homozygous for p.Met694Val.

**Figure 3:**
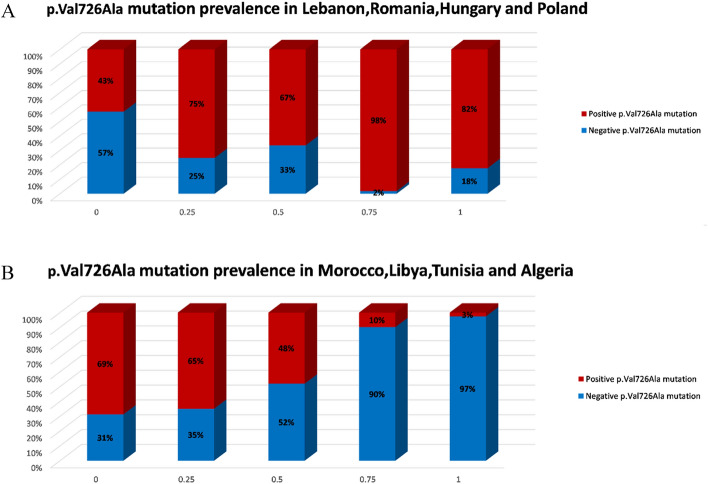
Prevalence of the countries of origin most indicative for the p.Val726Ala variant: (**A**) Lebanon, Romania, Hungary, and Poland are the countries of origin most positively associated with the p.Val726Ala variant. (**B**) Morocco, Libya, Tunisia, and Algeria are the countries of origin most negatively associated with the p.Val726Ala variant. Red and blue bars indicate patients who carry and do not carry the p.Val726Ala variant, respectively. The x-axis indicates the amount of grandparents from the four countries (0 being none, 1 being all four).

**Figure 4:**
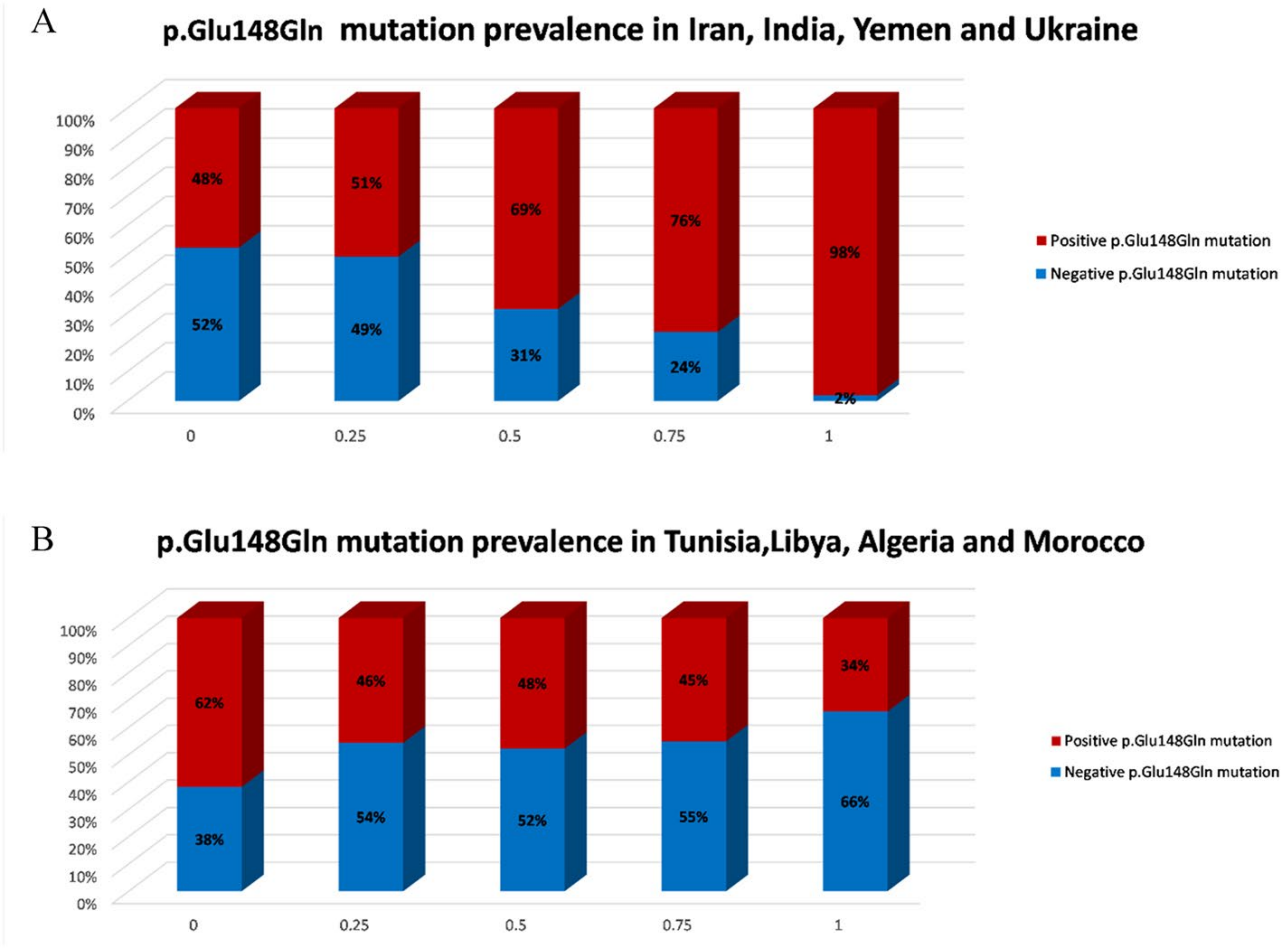
Prevalence of the countries of origin most indicative for the p.Glu148Gln variant: Countries that were found to be the most positively (**A**) and negatively (**B**) associated origins for a patient who carries a positive p.Glu148Gln variant**.** Red and blue bars represent the patients who carry and do not carry the p.Glu148Gln variant, respectively. The X-axis indicates the amount of grandparents from the four countries (0 being none, 1 being all four).

Origination in Tunisia, Libya, Morocco, and Algeria was positively associated with the p.Met694Val variant, roughly 70% referrals of Moroccan decent carried this variant. Origination in Romania, Germany, Iran and Poland reduced the chance of carrying the p.Met694Val variant (Fig. 1A, B). The performance of this prediction is demonstrated by the AUC of 0.78 (Fig. 2A). Moreover, by multivariate logistic regression analysis we demonstrated that Libya, Tunisia, Morocco and Algeria as countries of origin contributed the most to the probability that a referral would be homozygous for p.Met694Val variant with even higher degree of certainty (AUC = 0.86; Fig. 2D). Referrals with the p.Val726Ala variant had a high probability of originating from Lebanon, Romania, Hungary, or Poland (Fig. 3A). Ancestors from Morocco, Libya, Tunisia, and Algeria reduced the likelihood of this variant (Fig. 3B) with an AUC of 0.83 (Fig. 2B). Iran, India, Yemen and Ukraine are the origins that contribute the most to the existence of p.Glu148Gln variant (Fig. 4A), whereas Tunisia, Libya, Algeria and Morocco had an opposite impact (Fig. 4B) with an AUC of 0.67 (Fig. 2C). Fourteen of the studied countries did not show strong association with a single *MEFV* variant.

## Discussion

Many types of variants in the *MEFV* gene are associated with FMF. The five most commonly identified mutation types have been denoted as the founder mutations^7,8^. An association between ethnicity and the type of variant has been suggested, but a clear connection has not been established^17^. This study was conducted in order to demonstrate such link: We sought to demonstrate that a patient's variant could be predicted based on his/her family origins. Given the ancestral diversity of the large study population and the fact that the subjects rarely had a single country of origin, we constructed a model using machine learning in order to perform statistical analysis. We introduced an origin score to quantify the ethnic complexity of each individual.

Analysis of the study population, which included 1781 Israeli referrals for FMF testing mainly due to FMF suspicion, allowed us to extract reliable results despite the ethnic diversity of the study population. Our results showed that the p.Met694Val variant was the most prevalent among the study population, identified in 72% of studied subjects. Referrals whose parents or grandparents came to Israel from Tunisia, Libya, Algeria, or Morocco were most likely to carry this specific variant. The second most common mutation, p.Glu148Gln, was observed in 23% of the cohort; the most common countries of origin were Iran, Yemen, India, and Ukraine. The p.Val726Ala variant was the third most common, found in 19% of subjects. The countries of origin that mostly contributed to the existence of this variant are Lebanon, Romania, Hungary, and Poland. Notably, an inverse relationship exists between the p.Met694Val variant and p.Val726Ala with regards to country of origin. Countries that are most correlated with p.Met694Val are those least likely to be predictive of p.Val726Ala and vice versa.

All in all, the machine learning approach identified a single highly probable *MEFV* variant in 12 of the origins studied. The same was not the case for FMF referrals of other origins including Iraqi-Jews despite their high origin score, suggesting that at least two *MEFV* variants are probable in those origins. Based on the obtained results we deduce that the common *MEFV* variants in the Israeli Jewish population of our time have origins in a different geographical area: p.Met694Val in North Africa, p.Val726Ala in Europe and p.Glu148Gln in Asia. In general, the machine learning results are consistent with already established variants frequencies in North African, and Ashkenazi Jews^9^, yet they add a larger geographic scope and a better, country-wise perspective. For instance, our study identified Lebanon, a longtime residence of a small and relatively isolated Sephardi community, as the fourth country predicting Val726Ala, a variant considered to be of Ashkenazi origin (Ashkenazi allele frequency (AF) = 0.04, GnomAD^21^, https://gnomad.broadinstitute.org) (Table 2), perhaps as a consequence of random genetic drift. It is also intriguing that Ukraine, a residence of Ashkenazi Jewry, was found to be an origin of the p.Glu148Gln variant, along with a distant cluster of Asian countries. This finding could arise from the ethnic composition Ukraine immigrants to Israel, which includes mixed families of Ashkenazi and non-Jewish origins^22^. The geographical pattern of the p.Met694Val and p.Val726Ala variants observed in our study does not extend to the non-Jewish Caucasian populations of Europe (AF = 0.0009, gnomAD^21^) nor to the non-Jewish North-African population^23,24^, consistent with genetic drift, randomly occurring in small and isolated populations of the Jewish diaspora^9^, undergoing an evolution-based positive selection. Indeed a plague endemic could pose a rapid selection for *MEFV* variants introduced to Middle Eastern-derived populations early on^25^. Specifically the p.Met694Val and p.Val726Ala variants were shown to impede the evasion of *Yersinia pesti*s detection by the intracellular pathogen sensing system, which is mediated by the pyrin inflammasome^26^. Leukocytes from asymptomatic carriers mounted higher IL-1β levels in response to Y. pestis in *In-vitro* studies^25^, Emphasizing the selective advantage of *MEFV* heterozygotes.

**Table 2:** Frequency of the p.Met694Val , p.Val726Ala and p.glu148Gln variants in Jewish and corresponding geographic non-Jewish populations.

Ethnicity (healthy subjects)	Variant frequency p.Met694Val	Variant frequency p. Val726Ala	Variant frequency p. Glu148Gln	References
North African Jews in Israel (n = 100)	0.08	0	0.05	Stoffman et al.^29^
Ashkenazi Jews in Israel (n = 3472)	0	0.0425	0.05818	GnomAD V.3.1.2^21^
Iranian Jews in Israel (n = 100)	0	0	0.025	Stoffman et al.^29^
Yemen Jews in Israel (n = 36)	0.0138	0	0.0276	Feld et al.^30^
European—Non-Finish (n = 68,010)	0.0001764	0.0007205	0.01266	GnomAD V.3.1.2^21^
South Asians (n = 4828)	0	0	0.2981	GnomAD V.3.1.2^21^

Our study also identified an Asian origin for the p.Glu148Gln variant in the Israeli FMF referrals. An Asian origin is in agreement with the high frequency of this variant in the non-Jewish south and east Asian populations (AF = 0.298 and 0.280, respectively, GnomAD^21^), (Table 2). This observation may be rooted in the early settlement of the Asian Jewish diaspora and its admixture with the local population^27^. Considering that FMF *morbidity* is scarce in south Asian countries, the clinical significance of the p.Glu148Gln variant is uncertain, and it’s inclusion among the variants associated with FMF needs to be carefully discussed. A recent study showed a 17-fold increased penetrance of FMF in compound heterozygotes carrying both the p.Glu148Gln and p.Met694Val variants, over heterozygotes carrying the p. Met694Val variant alone, in north African Israeli-Jews^28^. This suggests that the p.Glu148Gln might be considered pathogenic in certain ethnicities.

The assignment of a certain variant to a particular origin might be compromised by two confounders: first, the study cohort mainly comprised symptomatic referrals, which may underrepresents low penetrance variants such as p.Val726Ala and p.Glu148Gln. However, the studied population concords with those served by practitioners, and therefore our results answer and are appropriate for medical needs. Second, the exclusion of compound heterozygous subjects could somewhat skew the results. However, the distribution of the excluded mutations is comparable to their distribution in the populations affected by this step. Therefore, its impact on the results is minimal.

## Supplementary Information

Supplementary Information.

## Data Availability

Upon request—From Prof. Shay.

## Code availability

Mentioned in the method section.
