# Supplementary material for: Determining the origin of different variants associated with familial mediterranean fever by machine-learning
Source: Sci Rep. 2022 Sep 8;12:15206. doi: 10.1038/s41598-022-19538-1 (PMC9458679; doi:10.1038/s41598-022-19538-1)
Supplement: Supplementary file 1 — Supplementary Information. [file 41598_2022_19538_MOESM1_ESM.docx]

**Determining the Origin of Different Variants Associated with Familial Mediterranean Fever by Machine-Learning**

Orit Adato^1,^*, Ronen Brenner^2,3^*, Avi Levy^1^, Yael Shinar^4^, Asaf Shemer^3,5,6^ , Shalem Dvir^5^ ,Ilan Ben-Zvi^3,7^ , Avi Livneh^3,4,7^ , Ron Unger^1,^* and Shaye Kivity ^3,8^*

^1^The Mina and Everard Goodman Faculty of Life Sciences, Bar-Ilan University, Ramat-Gan, Israel; ^2^Institute of Oncology, Wolfson Medical Center, Holon, Israel; ^3^Sackler Medical School, Tel Aviv University, Tel-Aviv, Israel; ^4^Israel Heller Institute of Medical Research, Sheba Medical Center, Tel Hashomer, Israel ^5^ Department of Medicine B, Assuta Ashdod Medical Center, Ashdod, Israel; ^6^ Department of Ophthalmology, Shamir Medical Center (formerly Assaf-Harofeh), Tzrifin, Israel;; ^7^ Department of Medicine F, Sheba Medical Center, Tel-Hashomer, Israel; ^8^ Rheumatology Unit, Meir Medical Center, Cfar-Saba, Israel

* Contributed equally

Address for correspondence:

Asaf Shemer MD

Department of Ophthalmology, Shamir Medical Center, Israel

Address: Be'er Ya'akov, 70300, Israel.

Phone: +972-8-9779620 Fax: +972-8-9779627 Email: ShemerAsafMD@gmail.com

**Supplementary Material**

**Figure S1**

Distribution of countries of origin by total sums of Origin Scores. Our analysis included the first 26 countries with the highest Origin Scores.


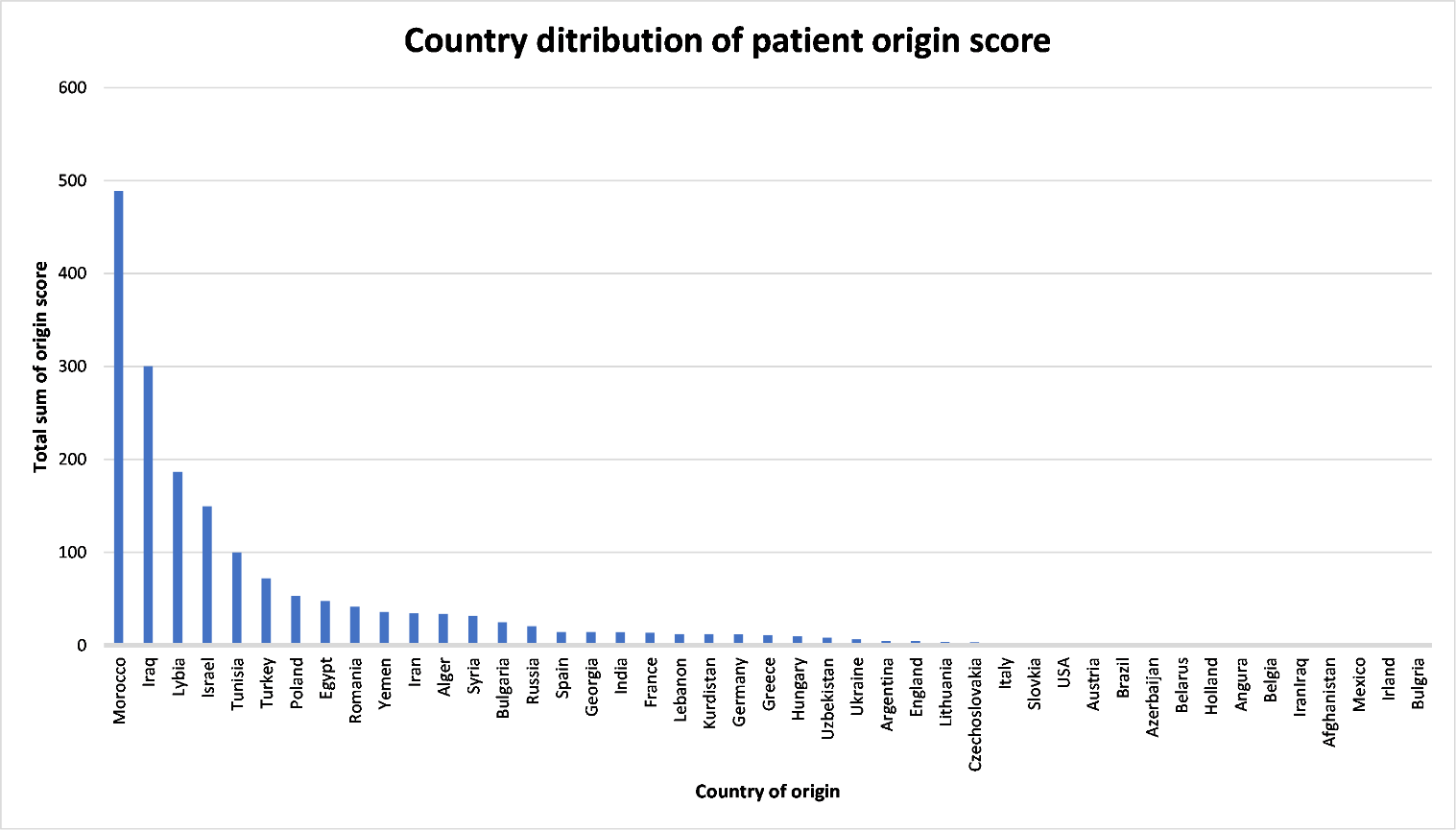


**Figure S2**

The distribution of different *MEFV* variant types among patients.

**
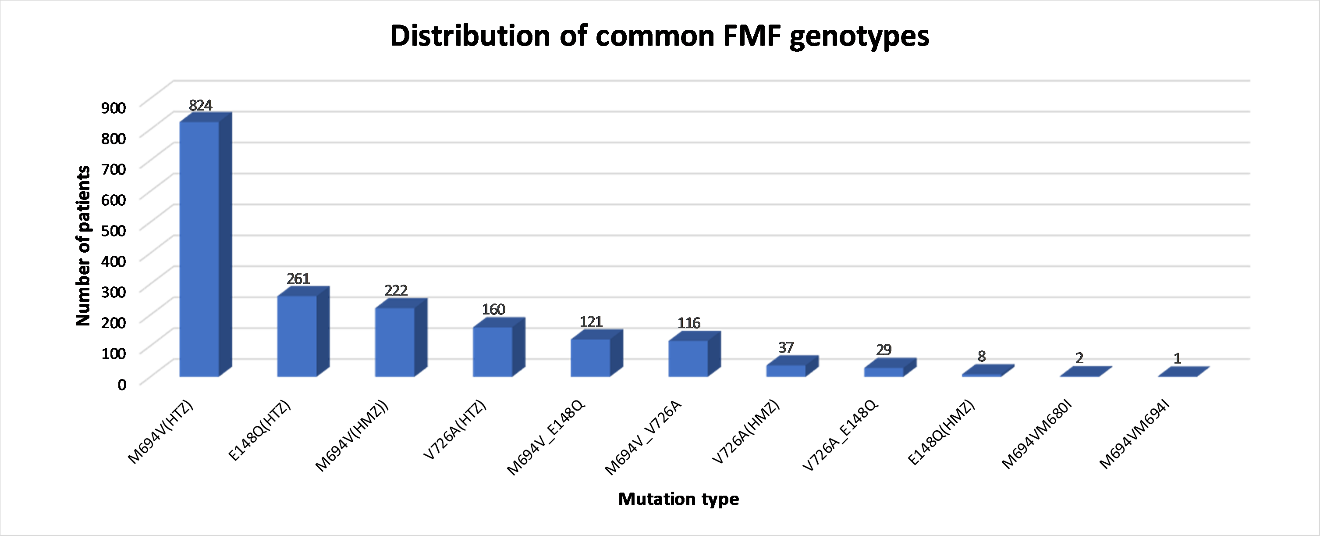
**

Figure S3:

**ROC of multivariate logistic regression for variant prediction- validated by bootstrap resampling**. The figure represents the performance of the multivariate logistic regression model using 27 features (26 countries and sex of the patient) to predict whether a patient: **A.** carries the variant p.Met694Val, **B.** carries the variant p.Val726Ala, **C**. carries the variant p.Glu148Gln, and **D.**  is homozygous for p.Met694Val. These ROCs display the results validated by bootstrap resampling 1000 times.


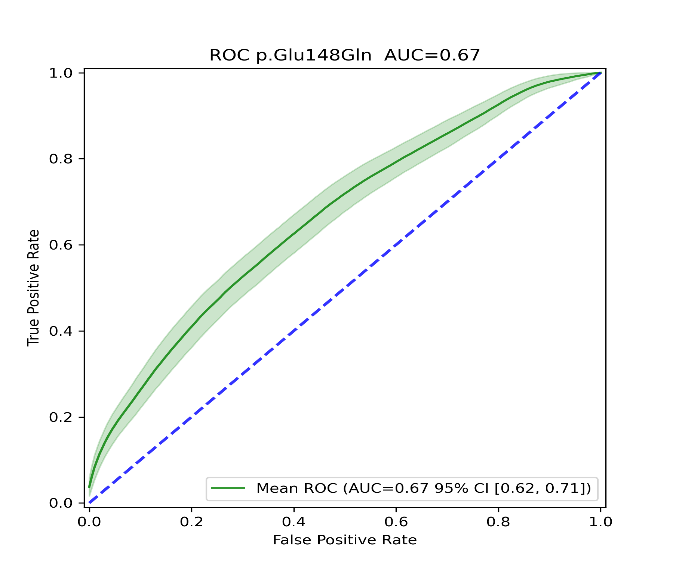

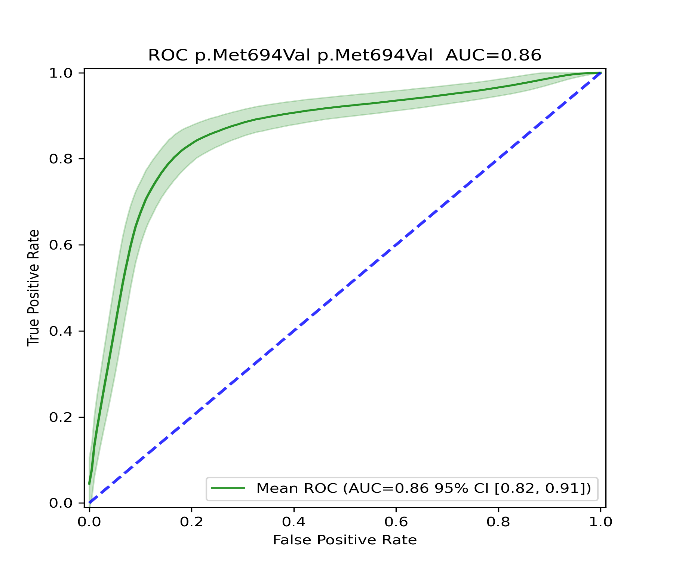

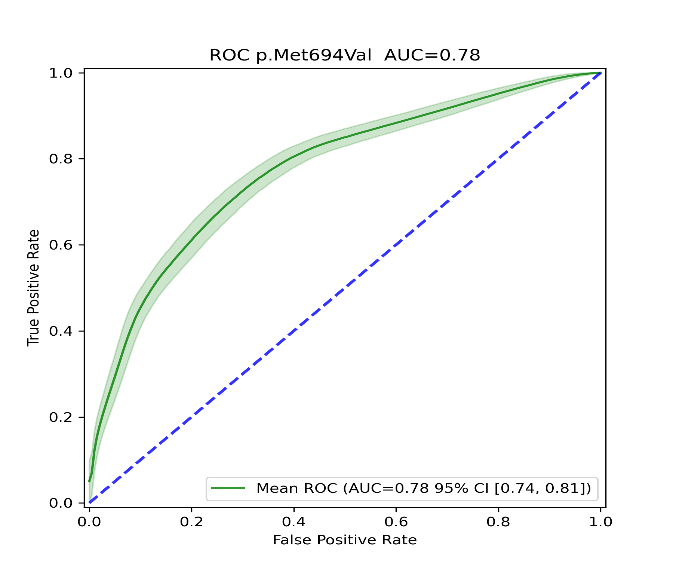

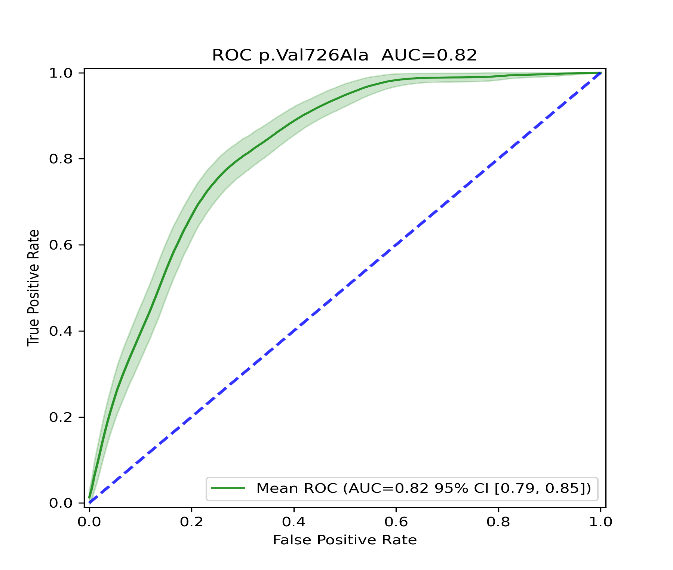


A

C

B

D

**METHODS – Supplementary Data**

Comparison with Bayes Theorem Calculation

Of the cohort of 1781 subjects, 600 patients (33%) had two parents or four grandparents with the same country of origin (origin score 1 for Country Group1, which includes Tunisia, Libya, Algeria, and Morocco). Technically, we can calculate the probability that a patient will carry variant p.Met694Val given that two parents or four grandparents were from country origin CountryGroup1 using Bayes rule as follows:

$$P\left( MUT1 \right)=0.56$$

$P\left( countryGroup1 | MUT1 \right)=0.39$

$$P\left( CountryGroup1 \right)=0.26$$

$$P\left( MUT1 | CountryGroup1 \right)=\frac{P\left( CountryGroup1 | MUT1 \right)*P(MUT1)}{P(CountryGroup1)}= \frac{0.39*0.56}{0.26}=0.84$$

Based on this calculation, the probability that a patient will carry p.Met694Valonly given that parents or grandparents all originated from countries of CountryGroup1 is 0.84. This number is identical to the value that resulted from the machine learning approach (the rightmost column of Figure 2). This validates our method. In addition, our machine learning algorithm also enables analyses of the associations between the country of origin and the variant along the entire spectrum of origin. For example, using our method, it is possible to predict whether a person will carry variant p.Met694Val based on his parents/grandparents origin with a AUC of 0.78 (Figure S2).
